# Implementation of the ‘Optimising the Health Extension Program’ Intervention in Ethiopia: A Process Evaluation Using Mixed Methods

**DOI:** 10.3390/ijerph17165803

**Published:** 2020-08-11

**Authors:** Yemisrach B. Okwaraji, Zelee Hill, Atkure Defar, Della Berhanu, Desta Wolassa, Lars Åke Persson, Geremew Gonfa, Joanna A. Schellenberg

**Affiliations:** 1 London School of Hygiene & Tropical Medicine, London WC1E 7HT, UK; della.berhanu@lshtm.ac.uk (D.B.); Lars.Persson@lshtm.ac.uk (L.Å.P.); Joanna.schellenberg@lshtm.ac.uk (J.A.S.); 2 Ethiopian Public Health Institute, Addis Ababa 5654, Ethiopia; atkuredefar@gmail.com (A.D.); desta.wolassa@lshtm.ac.uk (D.W.); Geremew2013@gmail.com (G.G.); 3 Institute for Global Health, University College London, London WC1E 6BT, UK; z.hill@ucl.ac.uk; 4 Institute of Public Health, College of Medicine and Health Science, University of Gondar, Gondar 41822, Ethiopia

**Keywords:** fidelity, care-seeking, complex community-based intervention, child health, behaviour

## Abstract

An intervention called ‘Optimising the Health Extension Program’, aiming to increase care-seeking for childhood illnesses in four regions of Ethiopia, was implemented between 2016 and 2018, and it included community engagement, capacity building, and district ownership and accountability. A pragmatic trial comparing 26 districts that received the intervention with 26 districts that did not found no evidence to suggest that the intervention increased utilisation of services. Here we used mixed methods to explore how the intervention was implemented. A fidelity analysis of each 31 intervention activities was performed, separately for the first phase and for the entire implementation period, to assess the extent to which what was planned was carried out. Qualitative interviews were undertaken with 39 implementers, to explore the successes and challenges of the implementation, and were analysed by using thematic analysis. Our findings show that the implementation was delayed, with only 19% (*n* = 6/31) activities having high fidelity in the first phase. Key challenges that presented barriers to timely implementation included the following: complexity both of the intervention itself and of administrative systems; inconsistent support from district health offices, partly due to competing priorities, such as the management of disease outbreaks; and infrequent supervision of health extension workers at the grassroots level. We conclude that, for sustainability, evidence-based interventions must be aligned with national health priorities and delivered within an existing health system. Strategies to overcome the resulting complexity include a realistic time frame and investment in district health teams, to support implementation at grassroots level.

## 1. Background

Despite major improvements in Ethiopia’s child mortality and substantial investments in child health programmes since the early 1990s [1,2,3], the utilisation of community-based child health services remains low across the country. For example, 31% of caregivers of children under five years of age sought care from an appropriate provider for pneumonia in 2016, as compared to 27% in 2011. Similarly, an estimated 30% of children under five years of age received oral rehydration salts treatment for diarrhoea from an appropriate health provider in 2016, as compared to 25% in 2011 [4,5]. Studies have reported that lack of geographic and financial access to services, caregivers’ limited knowledge of child health danger signs, and poor quality of care at health facilities were some of the reasons for this low utilisation of services [6,7,8,9,10]. 

**Figure 1:**
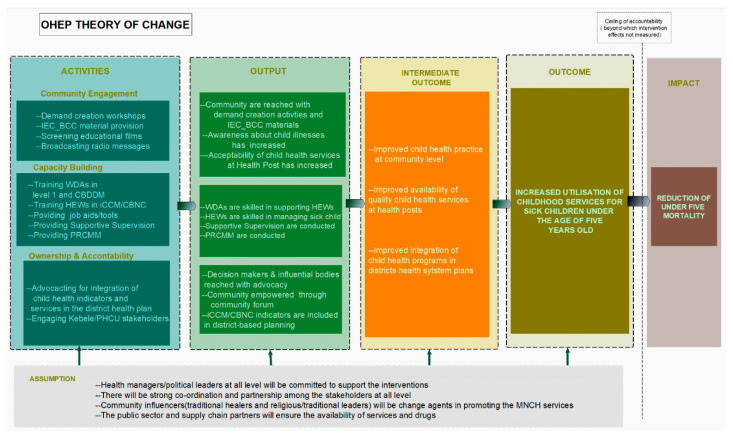
Simplified Theory of Change for the ‘Optimising the Health Extension Program’, to increase utilisation of childhood services in four regions of Ethiopia between 2016 and 2018. IEC_BCC— Information, Education, and Behaviour Change Communication; WDA—Women’s Development Army; CBDDM—Community-Based Data for Decision Making; HEWs—health extension workers; iCCM—integrated Community Case Management; CBNC—Community-Based New-Born Care; PRCMM—performance review and clinical monitoring meeting; PHCU—primary health-care unit.

Research suggests that interventions to improve care-seeking at the community level will be most successful if they are linked to the health system and target both supply- and demand-side issues [11,12,13,14]. Guided by these principles, the Ethiopian Ministry of Health, in partnership with four non-governmental organisations (NGOs), initiated a complex intervention to improve the utilisation of community-based child health services. The intervention, called ‘Optimising the Health Extension Programme’ (OHEP), was based on a theory of change which was designed by using formative research, including a barrier analysis and literature review [15] (Figure 1). This theory hypothesised the following: (1) through engagement in demand creation activities and exposure to Information Education and Communication or Behaviour Change Communication materials, caregivers would be knowledgeable about child health danger signs; be aware of availability of child health services, and would accept the quality of primary care; (2) through capacity building (training, supportive supervision, performance and clinical review meetings, and provision of job aids and tools and essential drugs for treatment of sick children) health extension worker (HEW) would be knowledgeable, skilled, and motivated in managing sick children; and (3) through advocacy for the inclusion of child health indicators in annual district plans and budgets and for the availability of ambulances for referral of severely ill children, districts would have improved ownership and accountability of child health programs. 

As a result of a synergistic effect of these three strategies, the overall utilisation of child health services for sick children was expected to increase. The effectiveness of the intervention was evaluated through a pragmatic trial comparing changes in care utilisation between 26 districts that received the intervention and 26 districts that did not [16,17]. The results from the trial showed no evidence to suggest that the OHEP intervention increased the utilisation of services for sick children from an appropriate health provider [17]. Here we assess the extent to which the components of the intervention were put in place as planned and explore the implementers’ perspective on successes and challenges of the implementation.

## 2. Methods

We took a mixed-method evaluation approach with concurrent data collection. The quantitative fidelity analyses explored the extent to which the intervention was implemented. The qualitative study used semi-structured interviews to gain a rich, in-depth understanding of implementers’ experiences of implementing the intervention as intended. The quantitative and qualitative data were conducted as complete components, and the data were analysed separately and then integrated for discussion. This approach ensures that the unique characteristics of each method are not lost.

### 2.1. Study Settings

The health-care delivery system in Ethiopia is broadly structured into primary, secondary, and tertiary health-care levels. The primary health-care unit (PHCU) is the lowest and most accessible point of service-delivery and provides basic health care. It constitutes five health posts linked to a single health centre. The health centres are linked with a primary hospital offering referral care at the district level. Each health post serves around 5000 residents and is staffed by two female health extension workers (HEWs) [2,18]. The OHEP intervention took place in the four most populous regions of Ethiopia, in 26 low-performing districts, where approximately 500,000 children under the age of five years reside. The low-performing districts were identified by the Regional Health Bureaux (RHBs), based on the level of utilisation of child health services at health centres and health-post levels. All health centres (*n* = 149), health posts (*n* = 675), and HEWs (*n* = 1604) in the 26 districts were targeted as the beneficiaries of the OHEP intervention. At the time of the baseline survey, which was conducted for the effectiveness study at the early stages of the implementation of the intervention, only 62% of health posts had water available, and only 18% had an electricity supply [19]. Shortages of essential drugs at health posts for treatment of childhood illnesses were also observed. For example, a third or more of the health posts did not have oral rehydration salts, and 57% did not have gentamicin. A fifth of the health posts had no zinc or amoxicillin. 

**Figure 2:**
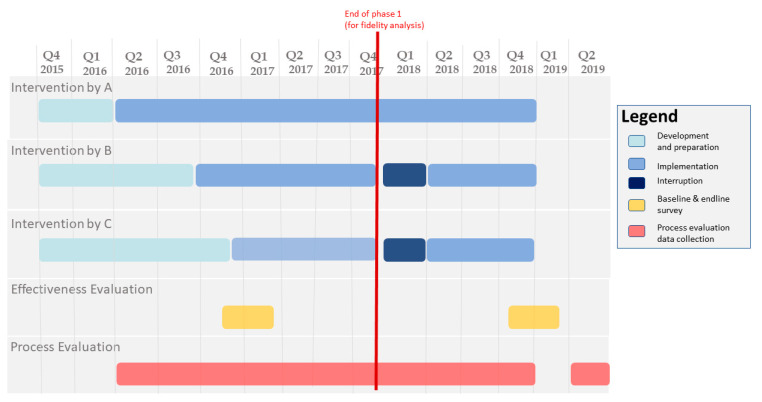
Timeline for OHEP intervention. Implementer A (A, B, and C are codes used to anonymise the three non-governmental-organization implementers) started implementation in March 2016, while Implementers B and C started in September 2016 and December 2016, respectively. The baseline and end line surveys were conducted in the last quarters of 2016 and 2018, respectively. The quantitative data for process evaluation were collected throughout the implementation period, while the qualitative data were collected in the 2nd quarter of 2019.

PATH (Program for Appropriate Technology in Health, a non-governmental organisation based in Ethiopia) implemented the OHEP intervention in Amhara and Oromia regions (17 districts) and UNICEF in Southern Nations, Nationalities, and Peoples (five districts) and Tigray regions (four districts). UNICEF subcontracted the implementation to Save the Children (Save the Children Ethiopia is a non-governmental organisation based in Ethiopia) and to the Last 10 Kilometres Project of John Snow International (Last 10 Kilometres Project of John Snow International is a non-governmental organisation based in Ethiopia). The intervention was implemented from March 2016 to December 2018, with the duration of implementation being different for each implementing NGOs (Figure 2). Implementer A (A, B, and C are codes used to anonymise the three NGO implementers) implemented for the entire 33-month period. Due to recruitment and other administrative issues, initiation was delayed by six months for implementer B and by nine months for implementer C. In addition, both had an interruption of implementation for four months, from December 2017 to March 2018. The delay and the interruption of the intervention are further explored in the qualitative findings and the discussion section.

### 2.2. Methods to Outline the Intervention Content

In order to make a detailed description of the intervention, we collected data on each intervention activity (Figure 1), between November 2017 and March 2018, using three different methods:

- Consultative meetings: We held consultative meetings with project staff and asked them to describe in detail what was delivered, with what aim, how much, to whom, by whom, and what mode of delivery. Notes from the meetings were collated into a narrative description of the intervention, which was verified by the implementers.
- Document review: We reviewed available program documents, including six implementation guidelines with training materials and procedure manuals. In addition, we reviewed four quarterly reports and over ten newsletters, to gather information about each intervention activity and its implementation plan. The documents we reviewed were in different formats, including hard copies and electronic documents. Some documents were in local languages, which were translated by the first author.
- Field visits: We made field visits to three intervention PHCU sites out of a total of 149 and spent two days in each site, with the goal of understanding the intervention and the implementation process. We observed implementation and reviewed procedure manuals and intervention materials available at the site. We conducted informal interviews with five to ten people in each site, including project staff and program participants such as HEW, schoolteachers, and district-level health staff, which were documented through informal field notes.

We used the Template for Intervention Description and Replication (TIDieR) checklist to generate an intervention description, using the data obtained from the three sources. TIDieR identifies 12 core items to describe the content of an intervention [20], including the purpose of each activity, what materials were used in the activity, who provided the activity, and how and where it was provided. We reported on all items from the TIDieR checklist (Supplementary Materials Table S3), except for those related to fidelity, as this was a key aim of the study and was reported separately. 

### 2.3. Quantitative Analysis of the Data 

We first reviewed documents on implementers’ monitoring and evaluation framework and their routine data collection tools, to see at what level implementation data were available and decide what fidelity data to extract and how to analyse them. The available data were for the implementation of district-level activities such as producing and delivering intervention materials. Although data for use and reach were documented at the community level, they were not collated at the district level and were not available to the study team. We split activities into two types: those which were one-time-only, such as an orientation workshop or production of educational films, and activities which were planned to continue over time, such as supportive supervision (Figure 1). 

Fidelity was defined as the extent to which activities were conducted according to plan and was measured by using implementation data [21,22]. To align with the effectiveness evaluation, which estimated impact for all intervention districts combined, our focus here was on fidelity for all districts. However, not all activities were planned for every one of the 26 intervention districts, and the results for each activity were restricted to those districts where it was planned. 

For each of 31 activities (Supplementary Materials Table S3), the overall fidelity in each district was calculated as a proportion, e.g., the number of workshops performed divided by the number planned. The list of fidelity indicators is shown in Table 1. The median and inter-quartile range of these proportions was then calculated for each activity across all 26 districts. To capture the timeliness of implementation, fidelity was calculated separately for the first phase (defined as the time from the start of the intervention until the end of 2017) and for the entire implementation period (Figure 2). We categorised median fidelity into three levels: less than 50%, 50–69%, and 70% or above. The threshold was created based on consultation with the implementers who recommended that 70% or above to be considered as high implementation, 50–69% as medium, and less than 50% as low. All quantitative analyses were conducted by using Stata 15 software (Stata Corp LLS, TX, USA).

### 2.4. Qualitative Methods

The qualitative data were collected through semi-structured interviews conducted between June and July 2019, four months after the completion of implementation. Interview topics were determined a priori, based on a literature review of similar studies and varied depending on the respondent type [23,24]. Topics included implementers’ overall impression of the intervention, what went well and what did not in implementing the intervention components, and the context within which the intervention was implemented. Respondents were not directly asked about reasons for any lack of effect, and at the time of the interview, respondents were not aware of the outcome of the effectiveness evaluation.

Data were collected from respondents at the head office of each of the implementing partners in Addis Ababa and from regional- and district-level respondents in intervention districts in Amhara, Oromia, Southern Nations, Nationalities, and Peoples and Tigray regions (Supplementary Materials Table S1). Purposive sampling was used to select respondents who had been involved in OHEP implementation for a minimum of six months. Respondents from implementing partners at the head-office and regional level who were already known to the researchers as being knowledgeable about the implementation were selected to be interviewed, and they were asked to identify government health staff for interview at the regional, zonal, and district level. At the national level, we interviewed NGO staff at head offices and Ministry of Health staff who had been involved in the design, planning, and high-level oversight of the intervention. In each region, we interviewed NGO and government-health-office staff who had been involved in planning and implementation, while at the zonal and district level, we interviewed NGO and health-system staff who were involved in the implementation. 

Supplementary Materials Table S2 shows the actual sample size (*n* = 39) for each respondent group, from a total of 54 planned interviews. The actual sample size was based on the concept of saturation sampling; that is, we continued investigating until no new information was elucidated [25]. Interviews were conducted face-to-face, in the appropriate local language, by five research assistants with a master’s degree and experience in qualitative interviews. The interviewers were trained for three days, and guides were pretested through role-play and pilot interviews outside the study area. Interviewers recorded all interviews and took field notes during the interviews. All interviews were fully transcribed and translated to English by two of the interviewers and two transcribers. The first author (Y.O.), together with the research coordinator (A.D.), ensured that the transcriptions, recordings, and translations were appropriately completed. During data collection, Y.O. compared a sample of twelve transcripts to the original recording and discussed with the interviewers, to ensure that emerging themes were followed up in subsequent interviews. 

A thematic analysis using a priori codes, themes, and subthemes that were most relevant to the research questions was developed using the interview guide topics [26]. During coding, we allowed additional themes to emerge. The analysis began with Y.O. identifying themes and sub-themes through a combination of manual and electronic coding and assigning the themes descriptive labels. A subset of transcripts was independently coded by Z.H., who also reviewed Y.O.’s coding on a regular basis. Discrepancies in coding were resolved through coding meetings, which were held several times during the analysis process. The codes were organised into broader themes, and the interviews were systematically indexed into categories and interpreted. The software Nvivo 12 (QSR International, Melbourne, Australia) was used both for data management and analysis.

The thirty-nine interviews included 49% (*n* = 19/39) government health office staff and 51% (*n* = 20/39) NGO staff. Of these, ten were involved in the design, planning, and high-level oversight of the intervention, while 74% (*n* = 29/39) were involved in implementation of the intervention. An estimated 92% (*n* = 36/39) of respondents had participated in the implementation for at least two years. The interviews lasted from 45 min to three hours. We report first on respondents’ perception of the intervention and its impact, followed by reporting of perceived challenging aspects of the implementation.

## 3. Results

### 3.1. The Intervention

Three strategies comprised the OHEP intervention: community engagement, capacity building, and ownership and accountability, with each strategy having several activities with a range of rationales. The detailed description of the intervention and the specific content of each activity based on the TIDieR checklist is provided in Supplementary Materials Table S3 [20]. Overall, there were 31 activities, of which 19 were community-engagement activities, eight capacity-building activities, and four ownership-and-accountability activities. Below, we describe each of these briefly, in turn.

The community-engagement strategy was designed to change community attitudes, norms, and behaviours toward care-seeking at primary health-care units. This involved engaging key community members, such as agricultural extension workers, religious and traditional leaders, and schools through demand creation workshops. In addition, this strategy included the provision of information, education, and behaviour-change communication materials to the community. This included a family health guide, posters, and brochures on danger signs during pregnancy and new-born care. Communities were also targeted through educational films and radio spots with messages promoting child health care and care-seeking for sick children.

The capacity-building strategy was designed to improve quality of services provided for common childhood illnesses at health posts and health centres. This involved (1) providing training for HEWs in new-born and child health programs, such as integrated Community Case Management and for the volunteer Women’s Development Army (WDA) leaders (level-one competency and Community Based Data for Decision Making) [27]; (2) improving skills of health workers, including HEWs, through supportive supervision, performance review, and clinical mentorship; and (3) providing HEWs with job aids, such as backpacks and foldable registration books, for case management of sick children during home visits. 

The ownership-and-accountability strategy was designed to improve district-level ownership of child health services. These involved NGO staff advocating for the integration of child-health-service indicators in the district’s health planning and management system, for standard opening hours of health posts, and for use of ambulances for referral of very sick children. This strategy also included strengthening the linkage between health centres and health posts so that health centres could provide supportive supervision and child-health-related drugs and supplies to health posts under their catchment area on a regular basis; engaging community stakeholders through a community forum, to discuss and voice their opinion on how to help improve services provided at the health posts; and NGO staff attending district annual planning meetings to support districts in the implementation of all the above activities in the district’s plan. 

### 3.2. Fidelity of Implementation 

From the 31 planned activities, 87% (*n* = 27/31) were one-time-only activities, and 13% (*n* = 4/31) were repeated activities (Supplementary Materials Table S3). Some of the one-time-only activities related to the delivery or production of items. For example, we measured the number of radio messages produced which were one-time only, but the radio broadcasting itself was a repeated activity. Table 1 presents fidelity results for activities under the community-engagement strategy, while Table 2 and Table 3 present fidelity results for activities under the capacity-building and ownership-and-accountability strategies, respectively.

Most activities were planned to be implemented early in the first phase, from the time each implementer started implementation to December 2017 (Figure 2). However, of all the 31 activities, only 19% (*n* = 6/31) had high fidelity in the first phase, with a median district fidelity of 70% or above. These were holding demand creation workshops and providing banners for health posts (Table 1), providing supportive supervision at health centres (Table 2), and conducting kebele and PHCU stakeholders’ workshops (Table 3). The majority of the activities 65% (*n* = 20/31) was implemented by the end of the implementation period. Late implementation occurred for health-post open houses; distribution of information and education print materials; production of radio messages and films; provision of projectors to health posts and Community Based Data for Decision Making training (Table 1); and provision of four of the five job aids and tools (Table 2). Some activities had low fidelity throughout the intervention period (median district fidelity less than 50%). This was the case for the provision of TVs and DVD sets for health centres and the provision of speaking books (Table 1). Similarly, training of HEWs in integrated Community Case Management and Community-Based New-Born Care programs and training of HEWs and WDA leaders in level one had less than 50% median district fidelity. Provision of supportive supervision for health posts and provision of linkage cards for WDA leaders to link with HEWs were also implemented with low fidelity (median district fidelity less than 50%) (Table 2). 

Among the four ownership-and-accountability activities, only conducting kebele and PHCU stakeholders’ workshops was achieved with high fidelity, in a timely way, with a median of 70% or more stakeholders participating in the workshops (Table 3). On the other hand, the other three activities, namely holding district-level advocacy workshop, conducting a community forum, and participating in district-level annual-based planning meeting, were achieved with a median district fidelity of 52%, 22%, and 33%, respectively, in the first phase (Table 3).

### 3.3. Perception of the Intervention and Its Impact

OHEP was described by both NGO and government health staff as an innovative intervention with a strong design. Respondents highlighted that the strong design was because intervention components were based on a review of literature, formative research to identify barriers and enablers, and consultation with experts and stakeholders. This formative research was followed by the development of a theory of change that showed the different components that should interact to bring about change. This view was summarised as follows:

*“One of the key factors for the success was developing the theory of change…all partners both from the government and non-government sectors working on maternal and child health were involved. So, having a proper theory of change helped us to understand what the project was going to implement”*

(NGO Staff 1 at the head office)

Respondents also reported the benefits of the intervention being developed by people with extensive experience in a range of maternal and child health initiatives in Ethiopia:

*“The selected implementing partners were well experienced, so they did not have any difficulty to implement the project. X and Y [names of implementing NGOs] have been working on child health for many years”*

(NGO Staff 2 at the head office)

In addition, some of the intervention activities were designed to be aligned with the primary targets of the Ministry of Health’s Health Sector Transformation Plan [2] and were implemented within the institutional structure of the health system.

*“What OHEP used was the existing structure. It has no parallel…The program was not about bringing change by creating a new structure. It was building capacity by employing new activities that were not dependant on modern technologies”*

(NGO Staff 3 at the head office)

In terms of the intervention’s impact, the general perception was that it had improved awareness in the community; for example, people were talking about child and new-born health and how sick children could be treated at health posts.

*“After the open house session people have known what is provided in at health posts, and that the HEWs and the services are there for the community, which has increased their interest in using health post services”*

(Government Staff 1 at Regional Health Bureau)

Quality of care was also perceived to have been improved both at health posts and health centres:

*“When we trained and supported them [HEWs], we saw that they had skill gaps. Thus, after training they knew more about their work and they showed a better performance and I think the change was very good”*

(Government Staff 2 at Regional Health Bureau)

### 3.4. Experience and Perception of Implementation Challenges

Implementation challenges were categorised into two main themes inspired by the realist evaluation approach [28]. The first theme focused on challenges related to the complex nature of the intervention itself, while the second theme focused on challenges related to the setting within which OHEP was implemented. This includes support and engagement of health managers, motivation of HEW, supervision on the ground, and external environment.

#### 3.4.1. Complexity

Although many respondents were enthusiastic about the comprehensiveness of the intervention, its complexity was perceived as a barrier for successful implementation, especially of the multicomponent nature and administrative challenges. 

##### Multiple Components

Document reviews and discussions with implementers emphasised the way in which many activities were interdependent. For example, all demand creation workshops with agricultural extension workers, schoolteachers, and religious or traditional leaders had to be completed before health-post open-house sessions could occur. Health-post open-house sessions were a key intervention component, as these showed the community what was available at the renovated health posts and connected the community with their HEW. As the workshops were held later than planned, so were the open-house sessions.

*“This is because it [open houses] needs the involvement of the community participation. It is at the community forum [workshops] that the date of the open house session is decided. The health post incurs costs to get renovation and needed the participation of the community, these things have taken some time”*

(NGO Staff 4 at district office)

An emerging theme indicated that OHEP attempted to do too much in too short a time. Respondents reported that one should not expect to observe the impact on behaviour unless all activities are put in place and given time to mature:

*“The duration of implementation was very short, and this kind of strategy needs time…. Convincing the community about their problem and convincing them that they can solve their problem is time taking. So, it needs time to introduce new idea and to let it mature but our strategy did not think of that”*

(NGO Staff 5 at the head office)

In addition, there were variations in the timing of implementing OHEP. For example, as can be seen in Figure 2, the timing with which sites progressed through the phases of implementation varied considerably, with two implementers having a late start date. A number of factors were consistently reported for causing the delay.

*“The first months were about hiring staff, equipping, and providing orientation about the project. The process of signing agreement takes time. And then at the end of the project, evaluation of the project is performed with the government. So, the duration of the implementation phase was short. When the second project [after interruption] started again, the process started all over again. There was lots of time wasted”*

(NGO Staff 6 at regional office)

##### Administrative Challenges

National-level NGO respondents reported difficulties in reaching a timely agreement between implementing and sub-contracting partners. This introduced administrative challenges in eight districts and led to an interruption for four months for the two of implementing partners, during which they laid off their staff and, thus, had to recruit new staff when activities resumed:

*“It shouldn’t have been interrupted because it was a hot time or peak time for the implementation. We were already delayed in starting the implementation”*

(NGO Staff 7 at head office)

Given the interruption and the initial delay, implementation was then rushed to make up time, and workers reported that they were very stretched:

*“I wouldn’t be exaggerating to say, we used to go out to the field the whole 11 months. We didn’t have breaks even on weekends. We were expected to complete a 2-year project within 1 year and 3 months”*

(NGO Staff 8 at district office)

Respondents also reported delays in procuring materials, e.g., materials for the speaking book. Instead of each implementer having its own supplier, respondents felt that having a single supply base for all implementers would have been more efficient.

*“If all partners could have purchased it from a single place, if the donor made the purchase process from a single place, it would have been fast. All have their own purchasing system and that was the reason for the delay”*

(NGO Staff 9 at the head office)

#### 3.4.2. Support and Engagement of Health Managers

One of the assumptions in the OHEP theory of change was that regional-, zonal-, and district-level health actors would be actively engaged and support the implementers. Good engagement was reported by a few respondents:

*“When other partners come, they discuss with the bureau and once they have started implementing, we only meet at the last review meeting. But with OHEP, we plan together, we train together and support the performance together”*

(Government Staff 1 at Regional Health Bureau)

Generally, the success of the implementation was largely dependent on whether districts prioritised their engagement in the intervention.

*“Most of the challenges when you work with government is their other competitive priorities, otherwise they were positive when they were working with us”*

(Government Staff 6 at district office)

In districts where the timing of implementation of OHEP activity coincided with districts priorities and where there were fewer disruptions due to health campaigns or disease outbreaks, more engagement of district health staff in the OHEP project was reported. However, overall, the dominant view was that engagement at the regional, zonal, and district level was not always achieved.

*“For instance, after we have planned certain activities to implement with health bureau, the plan will be postponed due to different reason such as trachoma campaign or immunisation campaign and so on”*

(Government Staff 9 at district office)

Further, it was perceived that ownership at the district level did not exist. OHEP, being perceived as a short-term donor-driven project, was frequently mentioned by respondents and summed up by two respondents:

*“The head of the district offices, directors of the health centres did not do follow up as if it [the program] was their own”*

(District Government Staff 6)

*“When they [health office staff] inquire about the project, they used to say, ‘What did you do with X’s [name of implementing NGO] project?’. They didn’t call the project as their own”*

(NGO Staff 3 at district office)

#### 3.4.3. Health Extension Workers as Catalytic Actors

HEWs were key actors in the implementation, as they work directly with the community. As such, the commitment and engagement of HEWs was seen by the respondents as essential for successful implementation at the grassroots level. However, implementers raised the issue of HEWs’ lack of commitment and engagement as hampering implementation.

*“I can say health extension workers have phased out; they don’t have motivation”*

(Government Staff 3 at Regional Health Bureau)

This led to a gap in implementation and delivery of activities on the ground.

*“But some extension workers do not work as asked. They train 30 women [WDAs] first and they were supposed to continue with the next 30 women, but they do not perform that, they stop with the first training. I’m still asking them to train”*

(Government Staff 2 at Regional Health Bureau)

#### 3.4.4. Shortage of Supervisory Staff and Transport on the Ground

Many of the activities implemented by the NGOs were focused at the district level. For example, the implementers delivered family health guides to district offices, with HEWs then required to deliver them to families. The implementers were required to supervise activities implemented by HEW at the kebele level. Respondents reported that their efforts to monitor if such activities occurred was constrained by shortage of supervisory staff on the ground.

*“There is a focal person that supports this program in each district but there should have been additional professionals on the ground”*

(NGO Staff 4 at district office)

Additionally, the lack of transport and fuel were reported as obstacles for monitoring ground level activities, as well as for effective implementation of some activities, such as supportive supervision:

*“When the health centre has a vehicle, they don’t have fuel. Or they need to have a motor bike, but they do not have one and even if they do, they don’t have fuel”*

(Government Staff 7 at district office)

Respondents felt that activities may have not always been implemented at the community level.

*“We deliver posters to the districts…the district may receive the materials but never distribute them”*

(NGO Staff 5 at district office)

#### 3.4.5. External Environment

When asked to reflect on external factors that may influence implementation, respondents consistently reported disruption caused by security issues and political instability.

*“The major challenge that we faced was instability. After we have done well then, the instability happens, so we leave”*

(NGO Staff 1 at head office)

Topography of the districts was also reported as a major implementation challenge:

*“Some districts are accessible, and others are very remote. It may take three or four hours walking to reach some health posts”*

(Government Staff 1 at head office)

Other external factors mentioned by NGO staff included competing priorities, such as supporting the management of disease outbreak diverting NGO staff from OHEP work, and thus compromising the timely implementation of the intervention:

*“When there is an outbreak the government forces us to focus on the outbreak or their focus will be diverted, and they will focus on the outbreak”*

(NGO Staff 8 at the head office)

## 4. Discussion

The literature from promotion and prevention programs reports a positive relationship between fidelity and intervention success. For example, in a review and meta-analysis of over 500 studies of promotion and preventive programs, Durlak and Dupre (2008) reported that the level of implementation achieved clearly affects program outcomes [29]. Although fidelity is considered to be the central component of implementation, there are also other components in the evaluation of implementation which could potentially affect intervention outcomes, such as transferability of evaluation findings into new contexts and adaptability [30]. However, these components are beyond the scope of this study.

In this study, implementation was delayed, with only one-fifth of activities having high fidelity in the first phase; however, most of the activities occurred later than planned. We identified key factors contributing to late implementation. Our fidelity results showed that there was a delay in implementation of some initial activities that would create the condition for the successful implementation of the next activity [31]. For instance, skills and knowledge of Community-Based Data for Decision Making tool was first supposed to be taught to HEWs who became trainers to train the WDAs. However, the training of trainers of HEWs was fully achieved toward the end of the project, leaving very little time for HEWs to cascade the training to WDAs. Similarly, projectors required for screening the films at health posts were mostly delivered in phase two, making it unlikely that all the target audiences in the community were reached through educational films. Thus, the intervention, which was complex, with multiple interactions between activities, had not fully taken place in a timely way and in the right sequence. 

Time was also needed for the intended audiences to contemplate the new ideas they were being introduced to and to be able to change their behaviour, as well as for that change to be detectable at the community level [32]. For example, caregivers’ awareness of availability of child health services was considered a prerequisite for behaviour change in care-seeking. As such, the immediate output of the interventions was to increase the proportion of caregivers who reported having seen or heard messages about available treatments for common childhood illnesses and increase the proportion of caregivers who were aware of the availability of child health services. However, in the baseline and post-intervention analysis, no improvement was found in either of these immediate outputs [17]. 

In addition to time, effective collaboration appeared to play an important role in achieving the project’s outcome. A scoping review of 25 published studies of collaboration partnership for health promotion across eight countries reported that the success of a project outcome could be a general indicator for how well partners are working together [33]. In this project, the collaboration brought together experts with a strong track record in designing community-based interventions. Despite this, our findings emphasised that administrative and logistic challenges brought additional complexity to the implementation, taking, for instance, collaborators having different supply bases for procurement or starting implementation at different time periods. Successful and timely implementation in all districts was necessary to achieve a final outcome. However, some of the districts experienced administrative challenges, which led to an implementation interruption and delay.

While the complex nature of the intervention was an important factor, favourable or unfavourable conditions in the settings within which the intervention was implemented also contributed to the implementation success [34]. First, in its design, the intervention recognised and included critical elements such as alignment to national health priorities and integration into the existing health system, and it had strong ownership at Ministry of Health level. However, alignment and integration at regional, zonal, and district health offices was anticipated to be achieved through the ownership-and-accountability activities. For example, one such activity was for NGO staff to participate in an annual planning meeting in every intervention district, in order to support the district technically and financially, to integrate key child health indicators and other measures in their annual plan and budget. As shown in the fidelity results, only one out of four ownership-and-accountability activities had high fidelity in the initial phase, and the intervention was seen as a supportive but separate donor-driven project, not as a program owned by the health system. This is likely to explain the reported inconsistent engagement of district-level ownership.

Second, the involvement of HEWs in cascading specific activities from higher levels to the grassroots level was central to the implementation success. However, respondents reported that HEWs were not adequately supervised and monitored and did not show adequate motivation to perform their expected activities. Consequently, the intended audiences at the community level may have not been reached as planned. We only have data from implementers’ perspective, and it would be beneficial to have data from the HEWs themselves. In other studies, HEWs’ lack of motivation has been due to a lack of skills and competing activities for which they were more closely monitored or supervised [35,36,37]. In line with this, a study that looked at implementation fidelity of initiatives to improve infant and young child feeding in Ethiopia reported that, while fidelity in the training of HEWs was high, gaps remained in cascading activities by HEWs to beneficiaries. The authors highlighted that, unless these gaps were addressed, it is unlikely that the expected impact at the household level will be observed [38]. In this project, the infrequent supervision of the HEWs may have contributed to HEWs’ lack of commitment and the low fidelity observed in activities cascaded by HEWs. For instance, the reported low fidelity in training of WDAs in level-one competency-based training could be a direct consequence of this.

## 5. Limitations

Our findings should be noted in light of the study’s limitations. First, our fidelity analysis focused on district-level activities such as producing and delivering intervention materials to districts. As such, cascaded activities implemented by HEWs at the lower level were not measured, despite being an essential part of the intervention. For example, the number of women HEWs had shown educational films to and the number of households that received the family health guide from HEWs were not recorded. Second, the preference would have been to collect the qualitative data during implementation, rather than after it stopped, but respondents were still able to recall implementation well, and the gap between implementation and data collection allowed respondents time to be more reflective of the intervention. Third, participation in the qualitative interview was limited to respondents who had been involved in the implementation of the intervention at district and higher level. It is likely that the HEWs experience of the implementation differed from those who participated in the interview. Last, there was a potential for bias in interpretation of the data due to the researchers’ knowledge of the intervention. However, in order to avoid this, we have considered all the data obtained, checked for consistency in a subset of coding between two researchers, and analysed it while continually re-evaluating the impressions and responses.

## 6. Conclusions

For sustainability, evidence-based interventions must be aligned with national health priorities and delivered within an existing health system. Strategies to overcome the resulting complexity include a realistic time frame and investment in district health teams to support implementation at the grassroots level. Furthermore, it was neither possible to identify what components were the essential ingredients nor the pathways through which change could have worked. We therefore recommend exploring the program theory of change and its assumptions to further assess whether the programme theory worked as planned.

## Figures and Tables

**Table 1:** Fidelity of implementation activities under Community Engagement Strategy in 26 districts categorised by implementation year (overall 2016–18; and restricted to 2016–17). IQR: inter-quartile range (fidelity benchmark: >70% pale green, 50–69% pale yellow, and <50% pale red).

Community Engagement	Activities	Activity Type: One Time Only?	Description	Indicator	No. of Districts That Planned Implementation	Phase 1 Implementation 2016–2017		Overall Implementation 2016–2018	
						Median	IQR	Median	IQR
Demand creation workshop	Agricultural Extension Workers (AEWs) workshop	Yes	AEWs (1330) to participate in orientation workshop	% of AEWs who participated in the workshop, per district	22 ^1^	88	58–100	95	71–100
	Schoolteachers’ workshop	Yes	Schoolteachers (929) to participate in orientation workshop	% of teachers who participated in the workshop, per district	26	100	100–100	100	100–100
	Religious/traditional leaders’ workshop	Yes	Religious/traditional leaders (115) to participate in workshop	% of religious/traditional leaders who participated in the workshop, per district	18 ^2^	100	75–100	100	75–100
	Health-post open-house session	Yes	Open-house session to be completed in 675 health posts	% of health posts which conducted open-house session, per district	26	6	0–49	96	77–100
IEC/BCC material provision	Family Health Guide	Yes	Family Health Guide (486,041) to be sent to districts for distribution toWDAs ^3^, HEWs, and families with under five children	% of Family Health Guide sent to districts for distribution to WDAs, HEWs and families with under five children, per district	26	46	1–73	81	72–100
	Brochure/factsheets	Yes	Brochure/factsheets (168,750) on childhood danger signs to be sent to districts for distribution to health posts, WDAs, Schools, and AEWs	% of brochure/factsheets sent to districts, for distribution to the community ^4^, per district	22 ^5^	43	17–61	89	81–100
	Posters for health facilities and the community	Yes	Posters (32,135) on childhood danger signs to be sent to districts for distribution WDAs, HEWS, AEWs, Schools	% of posters sent to districts for distribution to the community ^6^, per district	26	40	0–100	91	45–100
	Banners for health posts	Yes	Banners (675) to be sent to districts for distribution to health posts	% of banners sent to districts for distribution to health posts (assuming 1 for each health post), per district	26	92	50–100	100	93–100
	Pico projector for health posts	Yes	Pico projectors (675) to be sent to districts for distribution to health posts	% of health posts which received Pico projector (assuming 1 for each health posts), per district	26	0	0–83	100	100–100
	TV/DVD for health centres	Yes	TV/DVD (140) to be sent to districts for distribution to health centres	% health centres which received TV/DVD (assuming 1 for each health centre), per district	26	0	0–20	42	25–100
	Educational films production	Yes	Educational films (13) on new-born and pregnancy danger signs to be produced	% of locally appropriate educational films produced, per district	26	33	0–67	66	33–67
	Speaking book	Yes	Speaking book (486,041) with a sound and picture messages on maternal and child health to be sent to districts for distribution to families with under five children	% of speaking books sent to districts for distribution to households, per district	26	0	0–0	0	0–0
	Radio messages produced	Yes	Radio spots (25) on pregnancy, new-born, and child health to be produced for airing	% of radio spots in local language produced, per district	26	50	50–100	100	100–100
	Radio dramas produced	Yes	Radio dramas (2) on pregnancy, new-born, and child health to be produced for airing	% of radio dramas in local language produced, per district	18 ^7^	50	50–100	100	100–100

^1^ This activity was not planned to be implemented in 4 districts. ^2^ This activity was not planned to be implemented in 8 districts. ^3^ IEC/BCC: Information Education Communisation/Behaviour Change Communication. ^4^ Community includes AEWs, schools, WDAs, and HEWs. ^5^ This activity was not planned to be implemented in 4 districts. ^6^ Community includes AEWs, schoolteachers, WDAs, and HEWs. ^7^ This activity was not planned to be implemented in ^8^ districts where no budget was allocated for the activity.

**Table 2:** Fidelity of implementation activities under capacity-building strategy in 26 districts categorised by implementation year (overall 2016–2018; and restricted to 2016–2017). IQR: inter-quartile range (fidelity benchmark: >70% pale green, 50–69% pale yellow, and <50% pale red).

Capacity Building	Activities	Activity Type: One Time Only?	Description	Indicator	No. of Districts That Planned Implementation	Phase 1 Implementation 2016–2017		Overall Implementation 2016–2018	
						Median	IQR	Median	IQR
Training	iCCM/CBNC training for HEW	Yes	HEWs (1598) to be provided iCCM/CBNC ^8^ training	% of HEWs who participated in iCCM/CBNC training, per district	26	22	3–34	38	12–54
	Level-one competency training of trainers for HEW	Yes	HEWs (1598) to be provided level-one competency training of trainers training	% of HEWs who participated in level-one competency training of trainers training, per district	26	0	0–100	19	0–100
	Level-one competency for WDA	Yes	WDAs (22,593) to be provided level-one competency training	% of WDAs who participated in level-one competency training, per district	26	0	0–0	0	0–21
	CBDDM ^9^ training of trainers training for HEWs	Yes	HEWs (1598) to be provided CBDDM Training of trainers training	% of HEWs who participated in CBDDM training of trainers training, per district	22 ^10^	15	0–40	100	60–100
	CBDDM training for WDAs	Yes	WDAs (22,593) to be provided CBDDM training	% of WDAs who participated in CBDDM training, per district	22 ^11^	0	0–0	73	0–100
Joint supportive supervision	Joint supportive supervision for 50% health centres in the district	No	Joint supportive supervision to be provided to health centres	% of health centres which received joint supervision visits, per district (assuming 4 visits per year)	26	79	20–100	100	34–100
	Joint supportive supervision for 25% health posts in the district	No	Joint supportive supervision to be provided for health posts	% of health posts which received joint supervision visits, per district (assuming 4 visits per year)	26	16	12–21	40	29–48
	Performance review and clinical mentoring meeting (PRCMM)	No	PRCMM to be conducted for health posts at district level	% of PRCMM conducted, per district	26	2	1–12	75	35–100
Job aids and tools provision	WDA–HEW linkage card	Yes	WDA–HEW linkage cards (86,961) to be sent to Woreda, for distribution for WDAs	% of WDA–HEW linkage cards sent to district for distribution to WDAs per district	18 ^12^	0	0–0	13	0–100
	Backpack for HEWs	Yes	Backpacks (675) to be sent to district for distribution to HEWs	% of backpacks sent to district, for distribution to HEWs (assuming 1 per health post), per district	26	27	0–100	100	100–100
	Registration book (0–2)	Yes	Registration books (0–2) (675) to be sent to district for distribution to HEWs	% of registration books (0–2 months) sent to district, for distribution to health posts (assuming 1 per health posts), per district	18 ^13^	0	0–0	100	0–100
	Registration book (2–59)	Yes	Registration books (2–59) (675) to be sent to district for distribution to HEWs	% of registration books (2–59 months) sent to district, for distribution to health posts (assuming 1 per health post), per district	18 ^14^	0	0–0	100	0–100
	Chart booklet	Yes	Chart booklets (675) to be sent to district for distribution to health posts	% of chart booklets sent to district, for distribution to health posts (assuming 1 per health post), per district	18 ^15^	0	0–0	62	0–100

^8^ iCCM/CBNC—integrated Community-Based Management/Community-Based New-Born Care; ^9^ CBDDM—Community-Based Data for Decision-Making. ^10^ This activity was not planned to be implemented in four districts where the activity was already implemented through a previous project. ^11^ This activity was not planned to be implemented in four districts where the activity was already implemented through a previous project. ^12^ This activity was not planned to be implemented in eight districts where no budget was allocated for this activity. ^13^ This activity was not planned to be implemented in eight districts where no budget was allocated for this activity. ^14^ This activity was not planned to be implemented in eight districts where no budget was allocated for this activity. ^15^ This activity was not planned to be implemented in eight districts where no budget was allocated for this activity.

**Table 3:** Fidelity of implementation activities under ownership/accountability strategy in 26 districts categorised by implementation year (2016–2017 and 2018) IQR: inter-quartile range (fidelity benchmark: >70% pale green, 50–69% pale yellow, and <50% pale red).

Ownership and Accountability	Activities	Activity Type: One Time Only?	Description	Indicator	No. of Districts That Planned Implementation	Phase 1 Implementation 2016–2017		Overall Implementation 2016–2018	
						Median	IQR	Median	IQR
Ownership-and-accountability workshops	District-level advocacy workshop	Yes	District staff (1397) to participate in advocacy workshop	% of district staff who participated in advocacy workshop, per district	26	52	44–100	52	44–100
	Kebele/PHCU ^16^ stakeholders’ workshop	Yes	Kebele/PHCU stakeholders (2, 422) to participate in stakeholder workshop	% of stakeholders who participated in kebele/PHCU stakeholders’ workshop, per district	26	100	0–100	100	82–100
	Community forum	Yes	Stakeholders (12,034) to participate in the workshop	% of stakeholders participated in the workshop, per district	26	22	0–81	79	19–96
	Annual district-based Planning	No	Annual district-based planning sessions (66) to be attended by implementers	% of annual district-based planning session in which implementers participated, per district (assuming 1 participation per year)	26	33	33–67	67	67–100

^16^ PHCU—primary health-care unit.

## Data Availability

This study brought together implementation data obtained upon request from intervention implementers and a primary qualitative data. Due to confidentiality agreements with research collaborators, implementation data and interview transcripts cannot be made openly available.

## Supplementary Materials

The following are available online at https://www.mdpi.com/1660-4601/17/16/5803/s1. Table S1. Characteristics of study districts. Two districts were selected in Amhara and Oromia, as these regions contained the majority of intervention districts, and one district in each of the other regions. The districts were selected purposively to ensure heterogeneity in religion, ethnicity, and topography. Table S2. shows sample size of the interviewed respondents who were involved in the OHEP intervention. The project staff both from NGO and government health staff who participated in the implementation of the intervention were estimated roughly to be 160 in total. Of these, we interviewed 39 staff. Table S3. Intervention description—structured detailing of the activities adapted from Template for Intervention Description and Replication (TIDieR) framework.

## Trial registration

Current controlled trial ISRCTN12040912. https://doi.org/10.1186/ISRCTN12040912
