# Supplementary material for: Implementation of the ‘Optimising the Health Extension Program’ Intervention in Ethiopia: A Process Evaluation Using Mixed Methods
Source: Int J Environ Res Public Health. 2020 Aug 11;17(16):5803. doi: 10.3390/ijerph17165803 (PMC7459764; doi:10.3390/ijerph17165803)
Supplement: Supplementary file 1 [file ijerph-17-05803-s001.zip › Table S1 and S2_Revised.docx]

Table S1. Characteristics of study districts .Two districts were selected in Amhara and Oromia, as these regions contained a majority of intervention districts, and one district in each of the other regions. The districts were selected purposively to ensure heterogeneity in religion, ethnicity, and topography.

| Region | Zone | District | Predominant rReligion | Predominant Ethnicity | Society | Terrain |
| --- | --- | --- | --- | --- | --- | --- |
| Amhara | Awi | A | Orthodox Christian | Amhara | Agrarian | Flat |
|  | Awi | B | Orthodox Christian | Agew | Agrarian | Hilly |
| Oromia | W. Hararge | C | Muslim | Oromo | Agrarian | Hilly |
|  | Guji | D | Wakefeta | Oromo | Pastoralist | Flat |
| SNNP | Segen | E | Protestants | Derashe | Agrarian | Hilly |
| Tigray | SE Tigray | F | Orthodox Christian | Tigray | Agrarian | Hilly |

| Respondents | Amhara  Region | SNNP Region | Tigray  Region | Oromia Region | Addis Ababa | Total |
| --- | --- | --- | --- | --- | --- | --- |
| National- level health system and NGO staff |  |  |  |  | 10 | 10 |
| Regional- level health system respondent | 2 | 2 | 1 | 1 |  | 6 |
| Zonal- level health system respondent | 1 | 1 | *^[[1]](#footnote-1)^ | 1 |  | 3 |
| District- level health system respondent | 2 | 2 | 2 | 2 |  | 8 |
| Regional- level NGO implementers | 1 | 1 | 1 | 1 |  | 4 |
| Zonal- level NGO implementers | 1 | 1 | * 1 |  |  | 2 |
| District- level NGO implementers | 1 | 1 | 2 | 2 |  | 6 |
| Total planned interviews | **8** | **8** | **6** | **7** | 10 | **39** |

Table S2- shows sample size of interviewed respondents from each region, zone, and district who were involved on the OHEP intervention. We do not have an exact total number of project staff but we knew that there were two to four focal persons at each implementers’ head office in Addis Ababa, and one or two staff at each region, zonal and district level. This gives us an estimate of 8 region level staff who were responsible for supervising the implementation at zonal level; 10 zonal staff who were responsible for supervising the implementation at district level; and 52 staff at district level staff who were responsible for supervising and leading the implementation at community level. There were roughly equal number of government staff at each level who were the focal person for the project from the health system side. Based on these estimate the total number of project staff could be around 160. Of these, we interviewed 39.

1. There is no zonal health office in Tigray region [↑](#footnote-ref-1)
