# Supplementary material for: Implementation of the ‘Optimising the Health Extension Program’ Intervention in Ethiopia: A Process Evaluation Using Mixed Methods
Source: Int J Environ Res Public Health. 2020 Aug 11;17(16):5803. doi: 10.3390/ijerph17165803 (PMC7459764; doi:10.3390/ijerph17165803)
Supplement: Supplementary file 1 [file ijerph-17-05803-s001.zip › Table S3_InterventionDescription.docx]

## Table S3 Intervention description-structured detailing of the activities adapted from Template for Intervention Description and Replication (TIDieR) framework

| **Nr** | **Intervention Activities** | **Activity type:**  **one time only?** | **Target audience** | **Where, and how much** | **Implementers/ facilitators** | **What was the purpose/what material used/what procedure used** | **Variation in content and implementation** |
| --- | --- | --- | --- | --- | --- | --- | --- |
| **Community Engagement: Demand creation workshops** | | | | | | |  |
| 1 | Agricultural extension workers workshop | Yes | Agricultural extension workers | At district level. Conducted once | Project staff  Kebele managers  HEWs  HC^[[1]](#endnote-1)^ staffs district health office staffs | **Purpose:** to engage agricultural extension workers to reach male partners  **Materials:** OHEP Kebele level orientation guide (local language) flip charts, action plan template with local language  **Procedure:** facilitators discussed the issue with case scenario/example and agreed on action plan to be taken. | Only **Implementer A** and **Implementer C** implemented this activity (22 districts) |
| 2 | Religious/ traditional leaders’ workshop | Yes | Religious and traditional leaders | At district level. Conducted once | Project staff  Kebele managers  HEWs^[[2]](#endnote-2)^  HC staffs district health office staffs | **Purpose:** to engage religious and traditional leaders  **Materials:** OHEP Kebele level orientation guide (local language), flip charts, action plan template with local language  **Procedure:** facilitators discussed the issue with case scenario/example; and agreed on action plan to be taken. | Only **Implementer A** implemented this activity (18 districts) |
| 3 | School teachers’ workshop | Yes | School teachers and 5-8 grade school children | At district level. Conducted once | Project staff  Kebele managers  HEWs  HC staffs District health office staffs | **Purpose:** to engage school children in awareness of maternal and child health services so that they can go on to spread this information to their mother or family  **Materials:** OHEP Kebele level orientation guide (local language), flip charts, action plan template with local language  **Procedure:** facilitators discussed the issue with case scenario/examples; and agreed on action plan to be taken. | All implemented this activity (26districts) |
| 4 | HP^[[3]](#endnote-3)^ open house session | Yes | Community | At HP level  4 to 6 Hr  Conducted once. | Project staff  Kebele manager/ administrators  HEWs  HC staffs District health office staffs | **Purpose:** to introduce newborn and child health services available in the health post; to Inform the communiy of HEWs capacity to provide care and the advantages of care seeking from the health post.  **Materials:** as per OHEP open session guideline  **Procedure:** Presentation, role play, songs, dialogue, visit to HP and observation, Kebele Command Post orientation guide, banner poster, family health guide. | **Implementer A** used Pico projector to show videos. Also had open house reporting template, community minute confirmation form, quarter report (Nr. of HP and ~ of participants.) (26districts)  **Implementer C-** had 31 HCs and 152 HPs.  **Implementer B-** had 88 HPs. Had community minute confirmation form, Number of participants. |
| **Community Engagement IEC_BCC^[[4]](#endnote-4)^ material provision** | | | | | | |  |
| 5 | Distribute family health guides | Yes | Household with Pregnant women or mothers with < 2 years old child and WDAs | At HP/ Kebele level | District staff | **Purpose:** to improve knowledge and awareness at household and community level about maternal and child health services.  **Materials:** family health guide with 79 messages about maternal and child health services.  **Procedure:** Project team delivered the guide to districts; districts sent them to HCs and HCs to HEWs. The HEWs then distributed the material to WDA^[[5]](#endnote-5)^s and to pregnant women who came for ANC or during home visits. | **Implementer A-** had printed and distributed.  **Implementer C-** had printed and distributed. In addition, received some from UNICEF. (22 districts)  **Implementer B-** had printed and distributed. |
| 6 | Brochure/factsheet | Yes | Community | At HP level  Implementer C and Implementer A | District staff | **Purpose:** to improve knowledge and awareness including childhood danger signs messages at HH and community level about MNCH.  **Materials:** Brochure/factsheet with messages about MNCH taken from family health guide.  **Procedure:** Project team delivered the tools to districts; districts sent the material to HCs and HCs to HEWs. The HEWs then distributed the materials to WDAs and to pregnant women who came for ANC or during home visits | **Implementer A-** Poster, brochure and fact sheet (18 districts)  **Implementer C-** Poster only (4 districts)  **Implementer B-** Poster and brochure  (4 districts) |
| 7 | Poster | Yes | Community | At HP level  Implementer C & Implementer A | District staff | **Purpose:** to improve knowledge and awareness at HH and community level about MNCH.  **Materials:** Brochure/factsheet with messages about MNCH taken from family health guide.  **Procedure:** Project team delivered the tools to districts; districts sent the material to HCs and HCs to HEWs. The HEWs then distributed the material to WDAs and to pregnant women who came for ANC or during home visits. | **Implementer A-** Poster, brochure and fact sheet (18 districts)  **Implementer C-** Poster only (4 districts)  **Implementer B-** Poster and brochure  (4 districts) |
| 8 | Banners | Yes | Health posts | At HP level | Project staff | **Purpose: t**o be used for displaying services provided by HP during HP Open House session.  **Materials:** Brochure/factsheet with messages about MNCH taken from family health guide.  **Procedure:** Project team delivered the tools to districts; districts sent the material to HCs and HCs to HEWs. | All implemented this activity (26 districts) |
| 9 | Pico projectors | Yes | Health posts | At HP level | Project staff | **Purpose:** to educate pregnant women at pregnant women’s conference about ANC, PNC nutrition and essential newborn care through educational films.  **Materials:** Battery run Pico projector.  **Procedure:** Project team delivered the tools to districts; districts sent the Pico projector to HCs and HCs to HPs | **Implementer A-** bought Pico projector for HPs if no electricity and TV for HCs. (18 districts) |
| 10 | TV and DVD for HCs | Yes | Health centres | One TV and DVD set per HC | Project staff | **Purpose:** to educate pregnant women at maternity waiting homes and at pregnant women conference about ANC, PNC nutrition and essential newborn care.  **Materials:** TV/DVD to show educational films.  **Procedure:** Project team deliver the TV to districts; districts sends them to HCs | **Implementer C-** bought 5 TV- Not every maternity (4 districts) home has TV as some don’t have electricity.(4 districts)  **Implementer B-** Bought 26 TV and 26 DVD for 26 HC. (4 districts) |
| 11 | Educational films | Yes | Pregnant women,  Mothers with under 5 years old children, family member and caretaker. | At HC/ MWH^[[6]](#endnote-6)^ and Kebele level/ HP  Repeatedly | Midwifes at HC and HEWs at HP | **Purpose:** to improve knowledge about MNCH including newborn danger signs  **Materials:** Educational film in USB flash, TV or Pico projector  **Procedure:** Educational video shown, and discussion held with the midwives/HEW about the video message and summarised by the midwife or HEW. Video shown at maternity waiting rooms and at HC | **Implementer A-** Produced **6 films** both in Amharic and Oromofia using local people to film the story. Has a template to register how many have seen the video. (18 districts)  **Implementer C-** Produced **2 films** in 2 local languages using local people to film the story. Distributed the films in 31 USB flash discs to 31 HCs. (4 districts).  **Implementer B-** Produced **3 films** in Tigrigna using consultants and distributed 96 USB flashes to be shown at 96 HCs and HPs. (4 districts) |
| 12 | Distribution of speaking book | Yes | Families with under five children | One speaking book per household | Project staff | **Purpose:** to improve knowledge about MNCH including newborn danger signs  **Materials:** Speaking book with sound and picture messages about key maternal and child health issues  **Procedure:** Project team deliver the speaking book to districts; districts sends them to HCs and HPs | Was never distributed |
| 13 | Producing radio spot | Yes | Community | **Implementer A** and **Implementer C** at regional and community level  **Implementer B** - at regional level | Local radio station | **Purpose:** to improve awareness on MNCH services including danger signs and promote care seeking for sick new-born/child at HP  **Materials: d**eveloped short messages to be broadcasted at the local radio media  **Procedure:** Script was given to the media and media developed the messages and broadcast them | **Implementer A-** Broadcasted 4 radio spots twice a week for three months in local languages. Schools were provided with message to broadcast it on their mini media. (18 districts)  **Implementer B-** Broadcasted once a day for 6 months. Gave to HC supervisors to broadcast at HC and HP. (4 districts)  **Implementer C** – Broadcasted 5 different messages in 5 language, each for 1 month. (4 districts) |
| 14 | Producing radio dramas | Yes | Community | **Implementer A** regional and community level | Local radio station | **Purpose:** to improve awareness on MNCH services including danger signs and promote care seeking for sick new-born/child at HP  **Materials:** developed short drama to be broadcasted at the local radio media  **Procedure:** Script is given to the media and media develop the messages and broadcast them | **Implementer A-** Broadcasted 4 radio spots twice a week for three months in local languages. Schools were provided with message in flash to broadcast it on their mini media. Only Implementer A included radio drama in addition to radio message (18 districts) |
| **Capacity Building: Training** | | | | | | |  |
| 15 | iCCM/CBNC Training for /HEW | Yes | Health Extension Workers | At zonal level  8 days | ToT trained HW (both from Gov. and project team) | **Purpose:** to strengthen the capacity of HEWs in iCCM/CBNC services  **Materials:** presentation, field visit, Clinical practice, video show, Facilitator guide, Participants manual, Chart booklet, iCCM/ CBNC register and drugs. Mannequin, wall chart  **Procedure:** as per the national Ministry of Health iCCM/CBNC guideline | **Implementer C**- Didn’t do this in OHEP because they have already done it in CBNC project. (4 districts) |
| 16 | Level 1 competency ToT^[[7]](#endnote-7)^ training for HEW | Yes | Health Extension Workers | At district level | ToT trained HW (both from Gov. and project team) | **Purpose:** to equip HEWs with ToT of level 1 competency training so that they cascade the training to WDAs  **Materials:** level 1 competency facilitators tool  **Procedures:** level 1 competency facilitators tool presentation, group work. | All implemented this activity (26 districts) |
| 17 | Level 1 competency training for WDA leaders | Yes | Woman development army leaders | At kebele level for 52 days | ToT trained HEW | **Purpose:** to train WDA leaders on level 1 competency  **Materials:** flip chart, FHG, educational films  **Procedure:** presentation, discussion, group work | All implemented this activity (26 districts) |
| 18 | Training in CBDDM ^[[8]](#endnote-8)^ToT for HEWs | Yes | HEWs, PHCU staff, kebele managers, District HO  max 30 participants. | At District level  Two days | Project team and trained team from District HO | **Purpose:** to equip HEWs with CBBDM knowledge and to roll out the training to WDA leaders  **Materials:** Flip chart presentation, wall chart; stickers, birth notification card, prepared map and map register, WDA-HEW linkage card CBDDM tool  **Procedure:** CBDDM facilitators training of participants, on how to train WDA leaders on CBBDM. District invited HEWs and project staff arranged the workshop. | **Implementer A**- includes community referral linkage form and map register that include sick child linkage and follow up/.  **Implementer B**- CBDDM was implemented at Implementer B sites long before OHEP. So Implementer B only did 2 days review meeting. (4districts)  **Implementer C and Implementer A** completed this activity. |
| 19 | Training in CBDDM  for WDA leaders | Yes | WDA leaders | At kebele level  Two days  4 hours per day | ToT trained HEWs | **Purpose:** to train WDA leaders on how to implement and use CBDDM to map community service users and to link them to HP  **Materials:** CBDDM facilitators and CBDDM tool (stickers, flip charts, birth notification card, prepared map and map register, WDA-HEW linkage card)  **Procedure:** presentation, discussion, group work, demonstration on how to develop a map of the catchment area | **Implementer A**- includes community referral linkage form and map register that include sick child linkage and follow up. (18 districts)  **Implementer C and Implementer B** did not do these in OHEP as such activities already exist in their site.  (8 districts) |
| **Capacity building: Supportive Supervision** | | | | | | | |
| 20 | Joint supportive supervision at HC | No | Health centre staff | At HP and HC,  quarterly | Project staff in collaboration with Districts | **Purpose:** is to provide support to health workers at HC that will help them improve their technical skills.  **Materials:** as per standard MoH^[[9]](#endnote-9)^ Supportive supervision guideline  **Procedure:** A joint supervision visits together with District supervisors to HCs and review HWs work and provide feedback or on-job training. | All implemented this activity (26 districts) |
| 21 | Joint supportive supervision at HP | No | Health extension workers | At HP and HC, quarterly | Project staff in collaboration with Districts | **Purpose:** is to provide support to health extension workers at HP that will help them improve their technical skills.  **Materials:** as per standard MoH Supportive supervision guideline  **Procedure:** A joint supervision visits together with HC supervisors to HPs and review HEWs work and provide feedback or on-job training. | All implemented this activity (26 districts) |
| 22 | Performance review and clinical mentoring meeting (PRCMM^[[10]](#endnote-10)^) | No | Health extension workers | At District level  2 days  Bi annual | District HO in collaboration with partners. | **Purpose:** to review HEW’s work based on cases from iCCM and CBNC registers and provide clinical mentorship on how to register, assess and treat a sick child.  **Materials:** as per standard MoH PRCMM guideline  **Procedure:** HEWs’ performance checked based on actual cases from their iCCM/ CBNC register. If skill gap was identified, HEWs were provided refreshment training including clinical practice. | **Implementer A** performed this activity at PHCU level in some districts. (14 districts)  **Implementer B and Implementer C** did not have this activity in OHEP but only during CBNC project. (8 districts) |
| **Capacity building: Job Aids/Tools provision** | | | | | | | |
| 23 | Distribute  WDA-HEW linkage card |  | Woman development army leaders |  | Project staff | **Purpose:** to be used by WDA leaders for referring a sick newborn/child or a pregnant woman to HPs.  **Materials:** a colour coded card. For example, pink card to be used to refer a pregnant woman.  **Procedure:** Project team delivered the tool to districts; districts to HCs and HCs to HEWs and HEWs to WDA leaders. | Only **Implementer A** implemented this activity (18 districts) |
| 24 | Distribute backpack | Yes | Health extension workers | At HP/ Kebele level  One time | Project staff | **Purpose:** to strengthen HEW’s outreach services.  **Materials:** leather Backpack  **Procedure:** Project team delivered the tool to districts; districts to HCs and HCs to HPs | All implemented this activity (26 districts) |
| 25 | Distribute  (0-2months) registration book | Yes | Health extension workers | At HP/ Kebele level  One time | Project team | **Purpose:** to enhance HEW’s performance in assessing, classifying and treating sick new-born or child.  **Materials:** foldable (0-2months) registration book  **Procedure:** Project team delivered the tool to districts; districts to HCs and HCs to HPs | **Implementer A**- has included foldable iCCM/CBNC register. (18 districts)  **Implementer B and Implementer C** -delivered only backpack and had no budget for foldable iCCM/CBNC register. (4 districts)  **Implementer C and Implementer B**- have this already on their sites from CBNC project (8 districts) |
| 26 | Distribute  (2-59months) registration book | Yes | Health extension workers | At HP/ Kebele level  One time | Project team | **Purpose:** to enhance HEW’s performance in assessing, classifying and treating sick new-born or child.  **Materials:** foldable (2-59months) registration book  **Procedure:** Project team delivered the tool to districts; districts to HCs and HCs to HPs | **Implementer C and Implementer B**-already had in their sites for the CBNC project (8 districts) |
| 27 | Distribute chart booklet | Yes | Health extension workers | At HP/ Kebele level  One time | Project team | **Purpose:** to enhance HEW’s performance in assessing, classifying and treating sick new-born or child.  **Materials:** Chart booklet  **Procedure:** Project team delivered the tool to districts; districts to HCs and HCs to HPs | **Implementer C and Implementer B-** already had this in their sites for the CBNC project (8 districts) |
| **Ownership & Accountability** | | | | | | |  |
| 28 | Advocacy workshop | Yes | District cabinet and PHCU^[[11]](#endnote-11)^ staff | At District level  Once | Project team | **Purpose:** to engage district leaders to own and be accountable for iCCM/CBNC^[[12]](#endnote-12)^ programs.  **Materials:** OHEP District level guidline, fact sheet  **Procedure:** Presentation, discussion, and drafting action plan | All implemented this activity (26 districts) |
| 29 | Kebele/PHCU stakeholders’ workshop | Yes | kebele managers, PHCU directors and HEW supervisors | At District level  Once for 2 days | Project team and trained team from District Health Office | **Purpose:** to engage kebele and PHCU level leaders to own and be accountable for iCCM/CBNC program.  **Materials:** OHEP District level kebele command post orientation guideline, family health guide.  **Procedure:** Presentation, flipchart, groupwork including field practice on how to facilitate community forum; | All implemented this activity (26 districts) |
| 30 | Community forum | Yes | Key community stakeholders: religious leaders, School teachers AEW^[[13]](#endnote-13)^, HEWs | At HP/ Kebele level  Once | Trained team (Kebele manager, KCP^[[14]](#endnote-14)^) | **Purpose:** to engage stakeholders with key role in the community to have a say or share their opinion that will help improve services provided at HP  **Materials:** OHEP Kebele level orientation guideline, banner, FHG, action plan template.  **Procedures:** facilitators discuss the issue with the community including case scenario; and agree on action plan to be taken by the community. | **Implementer C**- has merged this activity with HP open house. It was done the same day. (4 districts)  **Implementer B-** has information for 26 kebeles. |
| 31 | Annual District Based planning | No | District cabinet | Once per year at district level | Project staff | **Purpose:** to participate in annual District based planning to support districts to include key maternal and child health services in their plan  **Materials:** Performance update on OHEP activities  **Procedures:** attend the annual meeting and discuss with the District cabinet and agree on action plan. | All implemented this activity (26 districts)  . |

1. Health Centre [↑](#endnote-ref-1)
2. Health Extension Workers [↑](#endnote-ref-2)
3. Health Post [↑](#endnote-ref-3)
4. Information Education Communication/Behaviour Change Communication [↑](#endnote-ref-4)
5. Woman Development Army [↑](#endnote-ref-5)
6. Maternity Waiting Home [↑](#endnote-ref-6)
7. Training of Trainers [↑](#endnote-ref-7)
8. Community Based Data Decision Making [↑](#endnote-ref-8)
9. Ministry of Health [↑](#endnote-ref-9)
10. Performance Review and Clinical Mentoring Meeting [↑](#endnote-ref-10)
11. Primary Health Care Unit [↑](#endnote-ref-11)
12. Integrated Community Case Management/Community Based Newborn Care [↑](#endnote-ref-12)
13. Agricultural Extension Worker [↑](#endnote-ref-13)
14. Kebele Command Post [↑](#endnote-ref-14)
